# Essential Oils in the Alternative Control of Postharvest Diseases in Strawberries: Anthracnose and Gray Mold

**DOI:** 10.1021/acsomega.5c04305

**Published:** 2025-08-12

**Authors:** Marcia R. Pansera, Vitória M. M. Zuccoloto, Wendel P. Silvestre, Valdirene C. Sartori, Murilo C. Santos, Sandro Hilldebrand, Roselaine Facanali, Marcia Ortiz Mayo Marques, Gabriel F. Pauletti

**Affiliations:** 1 Postgraduate Program in Process Engineering and Technologies (PGEPROTEC). University of Caxias do Sul, Street Francisco Getúlio Vargas, 1130, Petrópolis, Caxias do Sul, RS 95070-560, Brazil; 2 Course of Agronomy. University of Caxias do Sul, Street Francisco Getúlio Vargas, 1130, Petrópolis, Caxias do Sul, RS 95070-560, Brazil; 3 Postgraduate Program in Biotechnology (PPGBIO). 58802 University of Caxias do Sul Street Francisco Getúlio Vargas, 1130, Petrópolis, Caxias do Sul, RS 95070-560, Brazil; 4 Agronomic Institute (IAC). Barão de Itapura Avenue, 1481, Botafogo, Campinas, SP 13075-630, Brazil

## Abstract

Strawberry (*Fragaria* × *ananassa*) is one of the most perishable fruits, requiring care from harvest to consumer arrival. Gray mold and anthracnose are the diseases that most affect this crop in the postharvest period. Essential oils have bioactive compounds that may help maintain fruit quality and retard fruit decay. This study aimed to evaluate the essential oils of *Cymbopogon citratus* (DC.) Stapf, *Rosmarinus officinalis* L., *Cymbopogon winterianus* Jowitt., *Melaleuca alternifolia* (Maiden & Betche) Cheel, and *Cinnamonum camphora* var. *linaloolifera* in the control of postharvest phytopathogens *Botrytis cinerea* and *Colletotrichum acutatum* in *in vitro* and *in vivo* assays. The essential oils were extracted by steam distillation and chemically analyzed (GC–MS). The antioxidant activity of the oils was evaluated by inhibiting DPPH^•^ and ABTS^•+^ radicals. The *in vitro* treatments were zero and polysorbate 0.20% v/v, 0.01%, 0.05%, 0.10%, 0.15, and 0.20% v/v of each essential oil, diluted in polysorbate 20 (1:1), using the PDA culture medium. The evaluations were performed by measuring the average diameter of the colonies at 3, 5, 7, 10, and 14 days. For the *in vivo* test, the treatments were sprayed on the fruits; the two best concentrations of the *in vitro* test were mixed with water and the surfactant polysorbate 20 (1:1). After incubation, disease severity was assessed visually using a diagrammatic scale. Firmness, pulp pH, soluble solid content, and titratable acidity were also assessed. The essential oils tested in this study demonstrated fungicidal effects on both phytopathogens, whose concentrations varied between 0.05% and 0.20% v/v. Regarding the antioxidant and antifungal effects, the essential oils of *C. citratus* and *C. winterianus* had the highest antioxidant capacity and the strongest antifungal effect, suggesting a potential link between these biological effects. The essential oil of *C. citratus* showed, at a concentration of 0.05% v/v, total control of the development of *B. cinerea* and *C. acutatum*. It also demonstrated better soluble solid content and titratable acidity, resulting in better fruit flavor, thus providing better productivity and quality of strawberry fruits.

## Graphical abstract

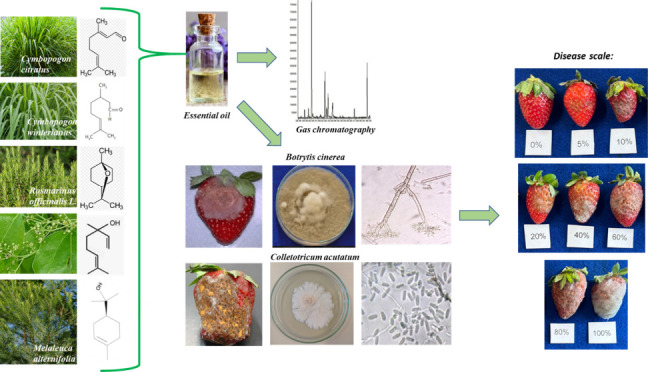

## 1 Introduction

The strawberry belongs to the Rosaceae family and is described as an herbaceous and perennial plant. There was a record production of strawberries in Brazil in 2018 with 3,481 t.^1^

The strawberry plant is susceptible to lack of water, low humidity, and high temperatures. It is considered a nonclimacteric fruit and is difficult to preserve due to rapid degradation caused by intense metabolic activity.^2^ Strawberries are rich in bioactive molecules, especially flavonoids, tannins, phenolic acids, and vitamin C, which have antioxidant activity.^3^

However, the high perishability of these fruits is often related to the incidence of rot, which directly affects the commercial product, causing qualitative and quantitative damage^4^ and reducing final productivity and quality. Among the primary diseases that cause losses in production in the field and postharvest of strawberries are anthracnose, caused by the *Colletotrichum acutatum* (Stoneman) Spauld. & H. Schrenk, and gray mold, caused by *Botrytis cinerea (Botryotinia cinerea* (De Bary) Whetzel, 1945).

Fungi utilize sophisticated penetration, infection, and colonization strategies to invade and suppress host defenses. Infection is initiated primarily by conidia that attach and germinate on the plant surface. Fungi can penetrate host tissues through natural openings such as stomata or wounds.^5,6^ These processes allow fungi to cause pre and postharvest losses by infecting all parts of their hosts, with organ choice dependent on environmental conditions.^7^

Control measures for these diseases are mainly based on preharvest spraying of chemical fungicides, and due to the great sensitivity of the fruits and their importance, these applications are repeated and intensified.^8^ In the search for new products with potential biological activity, much effort has been devoted to extracting and identifying natural products and secondary metabolites produced by plants, with essential oils being one of the most studied lines recently.

Essential oils are complex mixtures of volatile, lipophilic, low molecular weight, generally odorous and liquid mixtures consisting of terpenic molecules. In nature, essential oils protect plants as antibacterial, anti-inflammatory, antiviral, and antifungal agents and insecticides.^9^

In addition to being highly effective, essential oils have become an alternative to satisfy consumer demands for purchasing food products with fewer chemical additives or artificial inputs.^10^ In this sense, essential oils are alternatives to increase the shelf life of fruits due to their antimicrobial activity and low risk of developing resistance to pathogens, resulting from their complex composition and different mechanisms of action on target organisms.^11^

Essential oils are classified by the Food and Drug Administration (FDA) as safe for use in food, with growing interest in treating fruits and vegetables in the field and the postharvest and distribution stages.^12^

Although some studies have proven the toxic effect of essential oils on fungi, this work seeks to assess the effect of the chemical composition and antioxidant activity of *Cymbopogon citratus* (DC) Stapf, *Rosmarinus officinalis* L., *Cymbopogon winterianus* Jowitt., *Melaleuca alternifolia* (Maiden & Betche) Cheel, and *Cinnamomum camphora* var. *linaloolifera* essential oils and their antifungal effect *in vitro* and *in vivo* of *C. acutatum* and *B. cinerea* in strawberry fruits (*Fragaria* × *anasassa*), also evaluating fruit quality.

## 2 Results and Discussion

### 2.1 Analysis of the Chemical Composition of Essential Oils

The chemical composition of the essential oils of *C. citratus* (DC.) Stapf, *R. officinalis* L., *C. winterianus*., *M. alternifolia*, and *C. camphora* var. *linaloolifera* is presented in Table 1. The major components identified in *R. officinalis* were 1,8-cineole (27.27%), α-pinene (25.12%) and α-terpineol (8.26%). For the essential oil of *M. alternifolia,* the components were terpinen-4-ol (37.07%), terpinolene (17.27%), γ-terpinene (16.36%), and 1,8-cineole (9.86%). The essential oil from the leaves of *C. winterianus* presented citronellal (62.16%) and geraniol (7.06%).*C. citratus* presented the neral (34.77%) and geranial (48.77%), which together form citral (83.54%) and myrcene (10.89%), and the essential oil of *C. camphora* presented 96.73% of the compound linalool.

**Table 1:** Chemical Composition of the Essential Oils of *C. citratus*, *R. officinalis*, *C. winterianus*, *M. alternifolia*, and *C. camphora* var. *linaloolifera*, Extracted by Steam Distillation for 2 h^a^

			relative abundance (%)				
compound	LRI calc.	LRI lit.	*R. officinalis*	*M. alternifolia*	*C. winterianus*	*C. citratus*	*C. camphora*
α-thujene	927	924	0.18	0.22			
α-pinene	936	932	25.12	0.93	0.02		0.04
camphene	955	946	2.08	0.01			0.02
sabinene	977	969	0.05	0.01			
β-pinene	984	974	1.82	0.18			0.04
myrcene	992	988	1.23	0.50		10.89	0.02
α-phellandrene	1012	1002	0.19	0.56			
α-terpinene	1022	1014	0.53	5.87			
*p*-cymene	1031	1022	0.41	4.18	0.02		0.02
limonene	1034	1024	3.03	1.25	3.44	0.05	0.04
β-phellandrene	1037	1025		0.22			
1,8-cineole	1038	1026	27,27	9.86	0.03		0.06
(*E*)-β-ocimene	1049	1044		0.02		0.09	0.04
bergamotene	1059	1051			0.09		
γ-terpinene	1063	1054	1.43	16.36			
terpinolene	1091	1086	1.03	17.27			
6,7-epoxymyrcene	1098	1090				0.18	
fenchone	1100	1086	0.26				
*p*-cymenene	1100	1089		0.11			
perillene	1105	1102				0.12	
linalool	1106	1095	2.89				96.73
*exo*-fenchol	1129	1118	0.06				
chrysanthenone	1133	1124	0.79				
*exo*-isocitral	1151	1140				0.05	
β-pinene oxide	1159	1154				0.21	
camphor	1160	1141	2.76				1.1
citronella	1160	1148			62.16		
*iso*-isopulegol	1168	1155			2.62		
(*Z*)-isocitral	1169	1160				0.17	
*trans*-pinocamphone	1173	1158	0.07				
pinocarvone	1175	1160	0.33				
*iso*-menthone	1176	1158			0.05		
*neo*-isopulegol	1180	1167			0.10		
δ-terpineol	1181	1162	0.21	0.01	0.04		
borneol	1184	1165	3.71			0.07	
(*E*)-isocitral	1188	1177				0.43	
*cis*-pinocamphone	1189	1172	0.60				
terpinen-4-ol	1191	1174	1.32	37.07			0.02
*p*-cymen-8-ol	1192	1174		0.20			
α-terpineol	1206	1186	2.60	2.27			0.04
verbenone	1221	1204	8.26				
nerol	1234	1227	0.06				
citronellol	1234	1223			4.50	0.07	
neral	1249	1235				34.77	
carvone	1258	1239		0.02			
geraniol	1258	1249	3.53		7.06	1.12	
geranial	1279	1264	0.09		0.34	48.77	
bornyl acetate	1292	1284	2.66				
citronellyl acetate	1363	1350			2.65		
eugenol	1371	1356			0.04		
α-ylangene	1380	1373					0.04
geranyl acetate	1384	1379	0.16		2.15	0.13	
β-bourbonene	1391	1387			0.14		
β-elemene	1395	1389			1.39		
α-gurjunene	1411	1409		0.10			
(*E*)-caryophyllene	1425	1714	2.77	0.08	0.05		0.78
*trans*-α-bergamotene	1436	1432				0.07	
aromadendrene	1444	1439		0.52			
α-humulene	1462	1452	0.23		0.05		0.18
allo-aromadendrene	1467	1458		0.12			
germacrene D	1488	1480			0.52		0.11
α- muurolene	1505	1500			0.38		
germacrene A	1515	1508			0.30		0.05
δ-cadinene	1525	1522		0.26	1.93		
*cis*-calamenene	1530	1528		0.13			
elemol	1558	1548			1.57		
spathulenol	1587	1577		0.02			
caryophyllene oxide	1593	1582					0.06
γ-eudesmol	1644	1630			0.14		
α-muurolol	1654	1644			0.09		
monoterpenes			37.10	47.70	3.48	11.03	0.22
oxygenated monoterpenes			54.81	49.45	77.15	84.89	97.95
sesquiterpenes			3.00	1.21	5.15	0.13	0.98
oxygenated sesquiterpenes			2.82	0.02	1.80	0.05	0.06
total identified			97.76	98.38	87.58	96.10	99.21

a LRI Calc.: LRI values calculated from retention times of *n*-alkanes on HP-5 column. LRI Lit.: values based on Adams.^53^ Relative percentages based on the area normalization method.

It was also possible to observe that the essential oils presented oxygenated monoterpenes as the leading chemical class, with contents of 49.45% for *M. alternifolia* and 97.95% for *C. camphora*. It can also be noted that the contents of other chemical classes were distinct among the essential oils; e.g., *M. alternifolia* presented higher contents of monoterpenes (47.70%). In comparison, the contents of sesquiterpenes of all the plants researched were much lower (≥5.0%) when compared to oxygenated monoterpenes.

In a study reported by Mwithiga,^10^ the presence of the main compounds in rosemary oil were α-pinene (29.80–34.34%), 1,8 cineol (27.15–30.26%), verbenone (7.63–8.14%) and geraniol (4.47–5.22%). The biological activity of *R. officinalis* essential oil is attributed to several molecules, mainly monoterpenes, such as 1,8-cineole, borneol, pinene, limonene, camphene, camphor, and mircene.^13,14^

The essential oil of *M. alternifolia* is rich in monoterpenes and is characterized by a strong odor. The chemical composition depends on the extraction method used and the cultivation region. The same authors reported the identification of 14 components, which represent 92.87% of the oil, with terpinene-4-ol (31.11%), γ-terpinene (25.30%) and α-terpinene (12.70%) being the main constituents, followed by 1,8-cineole (6.83%),*p*-cymene (4.23%), terpinolene (4.03%), limonene (2.50%), α*-*terpineol (2.35%), aromadendrene (1.75%), and δ-cadinene (1.41%).^15^

*C. winterianus* oil presented *cis*-geraniol (32.85%), β-linalool (29.33%), and citronellal (14.50%) as the main components.^1^ Oliveira,^16^ through techniques of scanning electron microscopy, observed structural changes on the surface of *Colletotrichum acutatum* when exposed to *C. winterianus* essential oil, such as superficial wrinkles in the fungal hyphae, in addition to flaking, distortion, and destruction, making them nonviable.

Carvalho,^17^ analyzed the essential oil of *C. citratus*, observing the presence of 23 compounds representing 90.6% of the oil composition. The main components were geranial (42.2%), neral (31.5%), and β-myrcene (7.5%). Geranyl acetate (4.3%) and isopulegol (1.4%) were found in lower levels.^18^

The quality of *C. citratus* is generally determined by its citral content. Citral consists of *cis* (geranial) and *trans* (neral) isomers. The authors report that these mixtures present a multifaceted diversity of chemical substances, each playing a specific role in contributing to the characteristic aromas and potential biological activities inherent in these oils.^19^

Bordin^20^ evaluated the biological activity via fumigation of essential oils of *Cinnamomum* and *Citrus* spp. and pure compounds against *Dermanyssus gallinae* (De Geer) (Acari: Dermanyssidae), in addition to toxicity to the nontarget organism *Beauveria bassiana* (Vuill.). The same authors evaluated the composition of the essential oils of *C. cassia* and *C. camphora* var. *linaloolifera,* identifying 98.59% and 99.75% of the components, respectively. For *C. cassia*, the main component was *trans*-cinnamaldehyde (84.21%); for *C. camphora* var. *linaloolifera*, the main compound was linalool (98.75%).

Based on the literature, it is important to note that essential oils’ chemical composition and chemotypes varied considerably relative to geographical location. Variations in the chemical composition of essential oils are mainly due to genetic influence, especially in the essential oil chemotype (populations with genetic variations). On the other hand, environmental factors can influence the essential oil yield and the presence of minor and/or trace compounds. This assessment is important in evaluating the biological activity of essential oils since different chemotypes and chemical composition variations can influence these biological properties.^21^

### 2.2 Evaluation of the Antioxidant Activity

The results of the essential oils’ antioxidant activity (DPPH^•^ and ABTS^•+^) are presented as a sweeping percentage of each radical and as an equivalent concentration of Trolox and are shown in Table 2.

**Table 2:** Results of Antioxidant Activity of *C. citratus* (DC.) Stapf, *R. officinalis* L., *C. winterianus*Jowitt., *M. alternifolia*, and *C. camphora* var. *linaloolifera* Essential Oils Based on the Inhibition of DPPH^•^ and ABTS^•+^ Radicals^a^

	ABTS ^•+^			DPPH^•^	
essential oil	sweeping (%)	equiv. Trolox mM)		sweeping (%)	equiv. Trolox (mM)
*M. alternifolia*	45.8 ± 0.92 b	0.34 ± 0.00 b		76.1 ± 0.49 b	0.34 ± 0.00 c
*C. citratus*	99.0 ± 0.08 a	0.68 ± 0.00 a		93.3 ± 0.04 a	0.59 ± 0.00 a
*R. officinalis*	91.5 ± 0.87 a	0.64 ± 0.00 a		82.3 ± 0.31 b	0.44 ± 0.00 b
*C. winterianus*	98.4 ± 0.14 a	0.68 ± 9.00 a		94.4 ± 0.17 a	0.61 ± 0.00 a
*C. camphora*	50.4 ± 6.74 b	037 ± 0.04 b		25.8 ± 1.73c	0.19 ± 0.01 d

a The means in column followed by the same lowercase letter are not statistically different by Tukey’s test at a 5% error probability. Source: Authors (2024).

The essential oils of *C. citratus* and *C. winterianus* showed high antioxidant capacity measured by the DPPH^•^ (94.4% and 93.3%, respectively) and ABTS^•+^ (99.0% and 98.4%, respectively) methods, differing significantly from other essential oils. According to Martins,^22^ citral has potent antioxidant, hepatoprotective, antidepressant, estrogenic, and anti-inflammatory effects and is an antioxidant that prevents oxidative damage and neutralizes free radicals. The antioxidant capabilities of citral can reduce tissue damage and oxidative stress caused by biotic and abiotic stress sources.^17^

The antioxidant evaluation showed similar activity percentages considering the comparison between species within the same radical in sweeping percentage and millimolar equivalents of Trolox, but different indices when comparing the methods.^23^ The antioxidant activity values for the DPPH ^•^ radical of both products were like those found by Senol,^24^ who evaluated the antioxidant activity and the inhibitory effect of cholinesterase *in vitro* of *C. sempervirens* using different solvents (acetone, ethanol, and ethyl acetate), obtaining sweeping percentages in the range of 47–77% for the DPPH ^•^ radical.

Clain^25^ evaluated the antioxidant activity of *C. nardus* (’Ceylon’ citronella) extracts from different extraction methods. These authors reported an antioxidant activity of 1744 ± 155 μmol_Trolox_·g^–1^ for the hydroethanolic extract.

The essential oils of *R. officinalis*, *M. alternifolia*, and *C. camphora* presented antioxidant capacity of 82.3%, 76.1%, and 25.6%, respectively, evaluated by the DPPH^•^ method, and there was no significant difference between *R. officinalis* and *M. alternifolia.* For the ABTS ^•+^ method, the observed values of antioxidant capacity were 91.5%, 45.8%, and 50.4%, respectively, and *M. alternifolia* and *C. camphora* did not differ from each other.

The antioxidant capacity of *R. officinalis essential oil* may decrease tissue damage and oxidative stress. In DPPH neutralization, ABTS, and ferric reducing antioxidant power (FRAP) assays, proanthocyanidins Phytochemicals (PAs) from leaves and branches of *R. officinalis* showed prominent antioxidant activity. Essential oils are composed of different bioactivators, functional groups, and polarities. Their antioxidant effects cannot be attributed to only one or some of their constituents.^24^ They observed that using maltodextrin as an encapsulant of sweet orange essential oil resulted in higher values of antioxidant activity against the DPPH^•^ radical.

The antioxidant activity observed in essential oils can be entirely attributed to the more potent activity of the major components of essential oils. Other essential oil components also exhibited DPPH radical scavenging action, even though phenolic compounds are typically recognized as the source of the most intense antioxidant activity.^25^

However, it is difficult to attribute the antioxidant activity to one or a few active volatile compounds of the essential oil since it is a complex mixture of different volatile compounds. Generally, the antioxidant activity of the whole essential oil shows better radical scavenging capacity than the individual components, indicating the possible synergistic interaction between the different components of essential oils, which cannot be simplified or neglected.^26^

### 2.3 In vitro Antifungal Activity of Essential Oils

It was possible to verify a significant difference between the doses of essential oils in the variables analyzed. The essential oils tested showed fungicidal activity from the concentration of 0.15% v/v on the mycelial growth of *Botrytis cinerea* (Table 3).

**Table 3:** Percentage Inhibition (PI) for *Botrytis cinerea* Fungus When Subjected to Different Doses of *C. citratus*, *C. winterianus*, *R. officinalis*, *M. alternifolia*, and *C. camphora* var. *linaloolifera* Essential Oils after 14 Days of Incubation^a^

	essential oil concentration (% v/v)						
essential oil	zero	polysorbate (0.20)	0.01	0.05	0.10	0.15	0.20
*C. citratus*	0 Ba	0 Ba	0 Ba	100 Aa	100 Aa	100 Aa	100 Aa
*C. winterianus*	0 Ba	0 Ba	0 Ba	0 Bb	100 Aa	100 Aa	100 Aa
*R. officinalis*	0 Ba	0 Ba	0 Ba	0 Bb	100 Aa	100 Aa	100 Aa
*M. alternifolia*	0 Ba	0 Ba	0 Ba	0 Bb	100 Aa	100 Aa	100 Aa
*C. camphora*	0 Ba	0 Ba	0 Ba	0 Bb	0 Bb	100 Aa	100 Aa

a Means followed by the same letter, capitalized between rows (concentration) and lowercase in columns (type of essential oil), do not differ from each other by the Tukey test at 5% probability (*p* ≤ 0.05). Coefficient of variation: 0.00. Standard deviation: 0.00.

The essential oil of *C. citratus* showed fungicidal potential at a dose of 0.05% v/v in controlling *B. cinerea*, as it differed significantly from the other oils but did not differ from the other concentrations, promoting complete (100%) inhibition.

The other oils tested showed 100% inhibition from 0.10% v/v, except for *C. camphora* essential oil, which inhibited the phytopathogen from 0.15% v/v. Figure 1 shows the mycelial growth of the phytopathogen at concentrations of 0.10% v/v and 0.20% v/v on the 14th day of evaluation. The tests performed with polysorbate PA (concentration 0.20% v/v) showed no influence on the results since the phytopathogen grew in 100% of the plates, like the growth observed in the control treatment (only distilled water).

**Figure 1:**
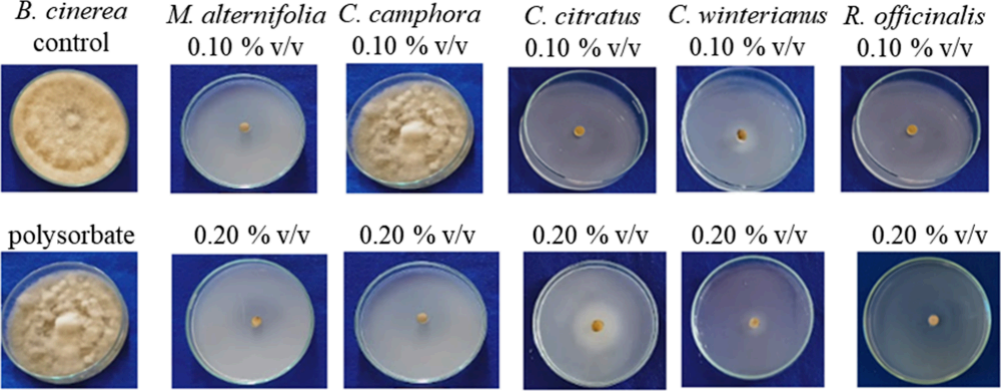
Results of the *in vitro test* with *Botrytis cinerea* using essential oils at concentrations of 0.10% and 0.20% v/v compared with the control and polysorbate (0.20% v/v) on the 14th day of mycelial growth.

According to the work carried out by Yan,^27^ the essential oils of *Cymbopogon citratus*, *Thymus vulgaris*, and *Origanum heracleoticum* exhibited maximum inhibition of the mycelial growth of *B. cinerea.* All three essential oils altered the hyphae’s morphology and ultrastructure, resulting in blisters along the hyphal surface. The essential oils damaged the plasma membrane of *B. cinerea cells* and leaked intercellular nucleic acids, proteins, and soluble sugars.

The essential oils of *Melaleuca*, *Cymbopogon citratus*, and *C. winterianus* showed a wide range of inhibitory activity against *B. cinerea*, depending on their species. At the same time, those of *Thymus*, *Pelargonium*, and *Origanum* exhibited intense inhibitory activity against *B. cinerea*, even at low concentrations,^28^ in the range of 50 μL·L^–1^.

Several studies confirm that some essential oils can damage the fungal membrane and promote electrolyte leakage, resulting in fungal death. Some essential oils have been reported to control plant diseases caused by various fungal pathogens, such as *Botrytis*, *Rhizopus*, *Penicillium*, *Alternaria,* and *Monilinia* in fresh fruits and vegetables.^29^

Linalool, present in the essential oil of *C. camphora,* exhibits antifungal activity against the fungus *B. cinerea*, downregulating ergosterol in the fungal cell membrane, impairing membrane integrity, damaging mitochondrial membranes, and decreasing ATP content.^30^ Linalool also inhibits pathogenicity by regulating plant defense responses and frequently interacts with other hormonal signaling pathways in vivo.^31^

In addition, linalool can also alleviate postharvest gray mold disease in tomato fruits by regulating the activities of antioxidant enzymes, secondary metabolism enzymes, and cell wall structure-related enzymes in tomato fruits. These results indicate that linalool is important in controlling tomato diseases.^32^

Table 4 presents the results of the tests carried out with the phytopathogen *Colletotrichum acutatum,* demonstrating a significant difference between the essential oils tested. The essential oil of *C. citratus* showed total inhibition of *C. acutatum* from a concentration of 0.05% v/v, and *C. winterianus oil* showed total inhibition from a concentration of 0.10% v/v, with no difference between them. The essential oils of *R. officinalis, M. alternifolia*, and *C. camphora* showed control of 15%, 13%, and 27%, respectively, at the highest concentration tested (0.20% v/v).

**Table 4:** Percentage of Inhibition Control (PIC) for the Fungus *Colletotrichum acutatum* When Subjected to Doses of *C. citratus* (DC) Stapf, *R. officinalis*, *C. winterianus*, *M. alternifolia*, and *V. camphora* var. *linaloolifera* Essential Oils after 14 Days of Incubation^a^

	essential oil concentrations (% v/v)						
essential oil	zero	polysorbate (0.20)	0.01	0.05	0.10	0.15	0.20
*C. citratus*	0.00 ± 0.00 Ca	0.00 ± 0.00 Ca	13.74 ± 1.87 Ba	100 ± 0.00 Aa	100 ± 0.00 Aa	100 ± 0.00Aa	100 ± 0.00Aa
*C. winterianus*	0.00 ± 0.00 Da	0.00 ± 0.00 Da	13.67 ± 6.09 Ca	36.14 ± 8.28 Bb	100 ± 0.00 Aa	100 ± 0.00 Aa	100 ± 0.00 Aa
*R. officinalis*	0.00 ± 0.00Ca	0.00 ± 0.00 Ca	5.03 ± 2.07 Bb	7.32 ± 1.56 Bd	9.67 ± 5.02 Bc	15.40 ± 1.97AAc	18.06 ± 2.59Ac
*M. alternifolia*	0.00 ± 0.00 Da	0.00 ± 0.00 Da	4.58 ± 2.88 Cb	6.85 ± 3.02 Cd	9.88 ± 3.50 Bc	13.99 ± 1.33 Bc	22.11 ± 2.53Ac
*C. camphora*	0.00 ± 0.00 Da	0.00 ± 0.00 Da	13.21 ± 1.45 Ca	15.65 ± 1.47 Cc	18.71 ± 2.08 Cb	27.57 ± 5.39 Bb	65.29 ± 2.84 Ab

a The value is the mean ± standard error of seven replicates. Means followed by the same letter, capitalized between rows (concentration) and lowercase in columns (type of essential oil) do not differ from each other by Tukey’s test at 5% probability of error (*p* ≤ 0.05). Coefficient of variation: 12.07%.

Figure 2 demonstrates the mycelial growth of the phytopathogen at concentrations of 0.10% and 0.20% v/v on the 14th day of evaluation. The tests performed with pure polysorbate (concentration 0.20% v/v) demonstrated no influence on the results, as there was total phytopathogen growth on all plates, like the control treatment (distilled water).

**Figure 2:**
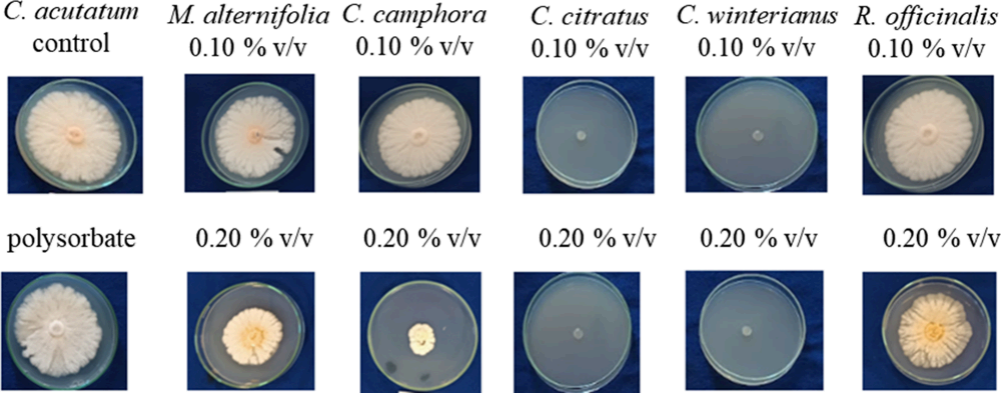
Results of the *in vitro test* with *C. acutatum* using essential oils at concentrations of 0.15% and 0.20% v/v compared with the control and polysorbate (0.20% v/v) on the 14th day of mycelial growth.

Anaruma^33^ demonstrated that 15 of 28 essential oils tested showed activity against *C. gloeosporioides*, and the following four oils showed MIC values between 0.25–0.30 mg·mL^–1^: *Coriandrum sativum, Cymbopogon citratus, C. flexuosus*, and *Lippia alba*. The evaluation of *C. citratus* essential oil in the control of postharvest rot in yellow passion fruit showed that the disease index of samples treated with the essential oil did not differ (*p* ≤ 0.05) from those treated with fungicide. The literature mentions that the essential oil can destroy the integrity of the fungal cell membrane, and the membrane structure can be altered. The cytoplasm of the fungal cell, including soluble protein, sugars, and nucleic acid, is released, altering the extracellular conductivity. For fungi, the integrity of the plasma membrane plays a crucial role in maintaining cellular constituents, which is essential for viability. Soluble sugar, protein, and nucleic acid are the primary and functional components of the cell.^34^

Wijesundara^35^ demonstrated that the mode of action for fungicidal activity may be linked to hydrophobicity and, consequently, to the ability of essential oil compounds to pass through the fungal cell membrane, which further affects pH homeostasis and inorganic ion balance, disrupting cellular structures. In addition, the involvement of the hydroxyl group (OH^–^) in hydrogen bond formation and the acidity of phenolic compounds may be other important factors involved in bioactivity.^36^

Several authors have evaluated the antimicrobial effect of the citral and geranial components present in *Cymbopogon* sp. on the horticultural pathogens *Rhizoctonia solani* (Ceratobasidiaceae), *Fusarium oxysporum* (Nectriaceae) and *Sclerotium rolfsii* (Atheliaceae).^37^ A variant of the poisoned food technique was used to verify the efficiency of these molecules and their selectivity according to the evaluation of phytopathogens. Citral was more effective in controlling *Fusarium oxysporum* (Nectriaceae), reaching 85% inhibition of radial mycelial growth with the addition of 0.4 μL·mL^–1^. At the same time, geranial was much more effective in controlling *S. rolfsii,* reaching 85% growth inhibition when 2.0 μL·mL^–1^ was used.^38^

This level of inhibition of individual molecules of *C. citratus* essential oil, such as citral, could suggest a possible synergistic effect of the 24 components identified in the essential oil in controlling the growth of *C. gloesporioides* since 100% inhibition of mycelial growth was achieved in this study.

It is important to highlight the higher antioxidant and antifungal activities of *C. citratus* and *C. winterianus* essential oils. Such a behavior may be linked to the presence of bioactive compounds, such as citronellal, geraniol, and neral, which are reported as having strong biological activity, including important antioxidant and antimicrobial effects.^39,40^

### 2.4 *In Vivo* Antifungal Activity of Essential Oils

The best concentrations of essential oils in the *in vitro* test (two concentrations of each oil) were used for the *in vivo tests.* In the case of the pathogen *Colletotrichum acutatum*, the essential oils that presented a control effect were *C. citratus* at 0.05% and 0.10% v/v and *C. winterianus* at 0.10% and 0.15% v/v, as shown in Table 5.

**Table 5:** Incidence (%) of the Phytopathogen *Colletotrichum acutatum* in Strawberries Treated with Increasing Concentrations of Essential Oils from *C. citratus* and *C. winterianus* ^a^

	essential oil	
concentration (% v/v)	*C. citratus*	*C. winterianus*
zero	86.0 ± 14.5 a	86.0 ± 14.5 a
0.05	3.7 ± 2.3 b	
0.10	0.0 ± 0.0 c	33.0 ± 17.6 b
0.15		10.0 ± 4.6 c
coefficient of variation (%)	30.55	36.72

a Means followed by the same letter in the column do not differ from each other by the Tukey test at a 5% probability of error.

The essential oil of *C. citratus* was shown to be effective in controlling postharvest strawberry anthracnose; i.e., at a concentration of 0.05% v/v, there was a 3.7% incidence of the disease, and at a concentration of 0.10% v/v, there was no disease. Regarding the essential oil of *C. winterianus*, there was an incidence of anthracnose of 33% at a concentration of 0.10% v/v and 10% at a concentration of 0.15% v/v.

In tests with *C. acutatum*, both *C. winterianus* and *C. citratus* essential oils have reduced the incidence compared to the control (without applying essential oils). The data regarding the incidence of *C. acutatum* in strawberries can be seen in Figure 3.

**Figure 3:**
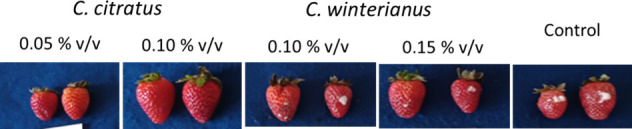
Results of the *in vivo* test with *C. acutatum* using essential oils at concentrations of 0.05% and 0.10% v/v for *C. citratus* and 0.10% and 0.15% v/v for *C. winterianus* compared to the control (no essential oil application).

Regarding the tests with *B. cinerea*, the essential oils that presented an *in vitro* control effect were *C. citratus* at 0.05% and 0.10% v/v, *C. winterianus* at 0.10% and 0.15% v/v, *M. alternifolia* at 0.10% and 0.15% v/v, *C. camphora* at 0.15% and 0.20% v/v and *R. officinalis* at 0.10% and 0.15% v/v. These were the concentrations used in the *in vivo tests.* The data from the *in vivo tests* with *B. cinerea* are shown in Table 6.

**Table 6:** Incidence (%) of *B. cinerea* in Strawberries Treated with Increasing Concentrations of Essential Oils from *C. citratus*, *C. winterianus*, *M. alternifolia*, *R. officinalis*, and *C. camphora* var. *linaloolifera* ^a^

	essential oil				
concentration (% v/v)	*C. citratus*	*C. winterianus*	*M. alternifolia*	*R. officinalis*	*C. camphora*
zero	90.0 ± 10.4 a	90.0 ± 10.4 a	90.0 ± 10.4 a	90.0 ± 10.4 a	90.0 ± 10.4 a
0.05	7.1 ± 4.5 b				
0.10	1.3 ± 2.2 c	13.3 ± 6.1 b	12.9 ± 15.4 b	18.8 ± 14.0 b	
0.15		10.8 ± 5.9 c	2.5 ± 2.6 b	14.2 ± 5.1 b	
0.20					11.3 ± 4.9 b
coefficient of variation (%)	20.42	20.49	31.29	25.66	19.70

a Means followed by the same letter in column do not differ from each other by the Tukey test at a 5% probability of error.

In the *in vivo* tests carried out with phytopathogen *B. cinerea*, all oils reduced the incidence of the disease compared to the control. The essential oils of *C. winterianus, M. alternifolia*, and *R. officinalis* were shown to be effective in controlling gray mold, with the best results at a concentration of 0.15% v/v (10.8%, 2.5%, and 14.2% disease incidence, respectively). Therefore, the essential oil of *C. citratus* was superior to the others, as it presented only 1.3% disease incidence at a concentration of 0.10% v/v. These results can be seen in Figure 4.

**Figure 4:**
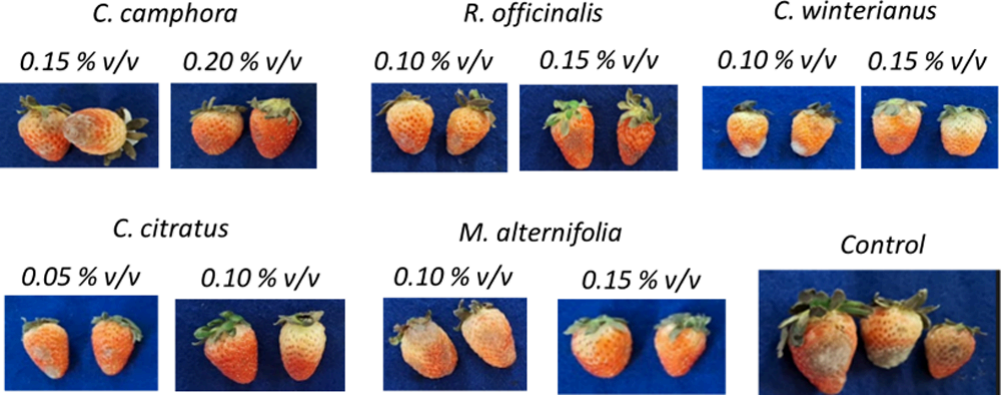
Results of the in vivo test with *B. cinerea* using the essential oils at different concentrations compared to the control.

The antifungal efficacy of essential oils was visually assessed using a diagrammatic scale from zero to 100% of fruits infected by phytopathogens relative to the total number of fruits in the sample. The incidence of fungal deterioration in strawberries was higher in the control group than in fruits coated with essential oils.^41^ Different concentrations of essential oils were efficient, reducing the incidence of diseases caused by *B. cinerea* and *C. acutatum*, both in curative treatment.

Pedrotti^42^ conducted tests with grapes treated with *Foeniculum vulgare* essential oil, demonstrating that the curative treatment was more efficient at a concentration of 200 μL·L^–1^, with no disease incidence detected. In the preventive treatment of *C. acutatum,* the concentration of 200 μL·L^–1^ inhibited the incidence of the disease, being different from the control. Similarly to the test with *B. cinerea*, the curative treatment of *C. acutatum* was more efficient, with concentrations of 50 μL·L^–1^ and 100 μL·L^–1^ significantly reducing the incidence of the disease. In contrast, the concentration of 200 μL·L^–1^ did not show any disease incidence. The severity of both diseases did not show any significant difference in the treatments with essential oil compared to the control when the disease was detected.

*B. cinerea* control treatments with essential oil reduced the lesion diameter for peaches without inoculation, with the essential oils *of C. winterianus*, *A. citriodora*, and *O. americanum* presenting the smallest averages, with 2.20 cm, 2.48 cm, and 2.51 cm, respectively, differing statistically from the control.

Both a significant reduction in microorganisms, deteriorating the quality of products and pathogens, and a close relationship between the dose of essential oil applied and the degree of inhibition of microbial growth are reported.^43^

*B. cinerea* is one of the most destructive fungal pathogens of fruits and vegetables, causing severe postharvest spoilage.^44^ According to Yan,^27^ exposure of strawberries to vapors of *Cymbopogon essential oils citratus*, *Thymus vulgraris*, and *Origanum heracleoticum* in commercial packaging reduced the incidence of gray mold, with *Thymus* and *Origanum* showing strong efficiency and reducing the disease rate by 53.85% and 57.69%, respectively. *In vivo experiments* demonstrated the inhibitory effect of these three essential oils on the gray mold of strawberries, suggesting these oils as potential alternatives to chemical fungicides in the management of postharvest decay of fruits and vegetables.

The authors showed that fennel essential oil affected the incidence of postharvest fungal rot in grapes caused by *B. cinerea* and *C. acutatum.* The essential oil inhibits postharvest pathogens, mainly due to its direct effect on the mycelial growth of the pathogens and the germination of conidia by affecting the cellular metabolism of the pathogens.^37^

Compared with the control, *F. vulgare* essential oil inhibited the growth of *B. cinerea* on plum fruits. *F. vulgare* essential oil has antifungal activity as a postharvest treatment against *B. cinerea* and *Penicillium expansum* in apples. The results showed that the antifungal activities of the essential oils were different under *in vitro* and *in vivo* conditions, and these activities were higher under *in vitro* conditions, requiring a higher concentration of the essential oil *in vivo.* The authors noted that these differences could be attributed to the alternation of the site of action of the essential oils or alternation in the fungal membranes under *in vivo* conditions.^45^

It is important to observe that, at up to 0.20% v/v, no signs of phytotoxicity were observed in the strawberries. However, it is advisable to avoid using greater concentrations since the essential oils at higher concentrations may have a toxic effect on the fruit’s tissues, promoting decay and decreasing the shelf life of the treated products.^16,38^

Relative to packaging and storage conditions, the exposure of essential oil to plastic containers or films may have a deleterious effect since the essential oil may tend to migrate to these plastics and even damage them. Considering the storage conditions, most perishable fruits, such as strawberry, are kept under slight refrigeration (5–10 °C), which is advantageous to retard essential oil volatilization and prolonging its presence in the fruit, keeping the antifungal and antioxidant effects for longer.

### 2.5 Effect of Selected Essential Oils on the Physical and Chemical Parameters of the Fruits

The physical and chemical parameters directly determine the technological utility of the fruit, as well as its flavor, storage, and organoleptic properties. Table 7 refers to the physical-chemical characteristics, namely firmness, pH, soluble solids, and titratable acidity of the fruits treated with increasing concentrations of essential oils of *C. citratus*, *C. winterianus, M. alternifolia, R. officinalis*, and *C. camphora* concerning the phytopathogen *B. cinerea* and *C. acutatum.*

**Table 7:** Firmness, pH, Soluble Solids (SS), and Titratable Acidity (TA) of Strawberry Fruits Treated with 0.10% v/v of the Essential Oils Tested Relative to *Botrytis cinerea* and *Colletotrichum acutatum* ^a^

	*Botrytis cinerea*			
treatment	firmness (N)	pH	TA (g·100 g^–1^)	SS (°Bx)
control (fruit without essential oil)	5.1 ± 1.4 a	3.0 b	8.06 a	5.26 c
*C. citratus*	3.7 ± 1.3 d	4.3 a	3.26 c	8.06 a
*C. winterianus*	3.9 ± 1.6 c	3.8 a	3.53 c	8.03 a
*M. alternifolia*	4.2 ± 1.7 c	4.0 a	4.56 b	6.57 b
*R. officinalis*	4.5 ± 1.1 b	3.8 a	4.16 b	8.03 a
coefficient of variation (%)	35.44	1.61	1.20	0.75
treatment	*Colletotrichum acutatum*			
control (fruit without essential oil)	4.7 ± 0.8 a	3.2 b	8.1 a	5.3c
*C. citratus*	2.9 ± 0.9 c	4.4 a	6.1 b	6.6 b
*C. winterianus*	3.9 ± 1.6 b	3.6 b	4.6 c	8.0 a
Coefficient of variation (%)	25.10	1.80	1.30	0.90

a Means followed by the same letter in the column do not differ from each other by Tukey’s test at 5% probability. AT - titratable acidity; SS - soluble solids. Titratable acidity is expressed in gram-equivalents of citric acid per 100 g of fruit. The standard deviations for pH, SS, and TA were less than 0.1 for all samples.

It can be observed that, for the evaluation of fruit firmness, all essential oils subjected to the control of both diseases promoted a reduction of these parameters, differing significantly relative to the control. Significant differences were observed between the control and the treatments tested. The control presented 5.1 N of firmness. The fruits exposed to the essential oil of *C. citratus* presented firmness of 3.7 N tested with *B. cinerea* and 2.9 N with *C. acutatum.*

Maintaining the firmness of the fruit pulp is an important parameter observed in the quality of postharvest management since it is associated with the best conservation conditions and visual appearance. Firmness is a complex parameter that can be influenced by several factors, including the internal structure of the fruit and its chemical composition.^46^ Firmness is a determining factor in the postharvest quality of strawberries, mainly because it affects the transportability of the fruit. The main reason for the loss of firmness (softening) of strawberries is the degradation of the cell wall, especially the middle lamella of the cells. Such behavior occurs mainly due to tissue damage caused by microorganisms such as fungi, but fruit respiration and water loss also contribute to cell wall degradation.^41^

The essential oil acts on the fruit’s cellular tissue, causing structural changes that lead to softening and increased release of enzymes or substrates, which favor this process. Carrillo^47^ observed that this softening was also observed in minimally processed melons with alginate-based coatings containing geraniol, a *Cymbopogom martinii* essential oil component.

Regarding pH, the oils submitted for control of *B. cinerea* increased the pH compared to the control. However, only the essential oil of *C. citratus* revealed an increase in pH when submitted for control of *C. acutatum.*

Determining the pH is an important parameter for the final destination of the fruits, with less acidic pH intended for consumption *in natura*. In comparison, those with more acidic pH are intended for industrialization. Microorganisms require specific pH, temperature, oxygen, nutrients, and humidity levels to develop; that is, depending on the acidity of the food, chemical reactions will occur.^48^ Postharvest reductions in the pH of strawberries occur mainly due to the action of fungi, which acidify the fruit as it decomposes, and consumers prefer strawberries with a low acidic pH; even these fruits tolerate a more acidic pH.^41^

Evaluating titratable acidity (TA) for all essential oils subjected to the control of both diseases promoted a reduction in this parameter, differing significantly concerning the control. The essential oils that presented the best titratable acidity values were *C. citratus* and *C. winterianus* 3.3 g·100 g^–1^ and 3.5 g·100 g^–1^, respectively, in tests with *B. cinerea.*

TA indicates the total quantity of acids present in strawberry juice (mainly ascorbic, malic, succinic, and citric acid) that contribute, together with soluble solids (SS), to define the flavor of the fruit, thus representing an important quality attribute.^48^

Regarding the SS evaluation, all essential oils subjected to the control of both diseases promoted an increase in this parameter, differing from the control. The strawberries treated with the essential oils of *C. citratus*, *C. winterianus*, and *R. officinalis* showed SS values between 8.1 °Bx and 8.0 °Bx, respectively.

For the strawberry fruit to have the best flavor, minimum values of 9.0 °Bx of SS and maximum values of 0.8 g·100 g^–1^ TA are required; i.e., the greater the soluble solids and the lower the titratable acidity of the fruit, the better its flavor will be.^47^

Sweetness is determined mainly by SS. The SS measurement is a good indicator of fruit ripeness. The compounds responsible for SS content, especially sugars, increase when the fruit remains on the plant. According to the Brazilian Program for the Modernization of Horticulture (PBMH), immature fruit is classified as a severe defect, defined by the minimum SS content that each fruit must have.^48^ Strawberries are considered poor when they have less than 6.0 °Bx, average for levels up to 10.0 °Bx, and excellent when the SS is equal to or greater than 16.0 °Bx.^49,50^

The observed SS values were higher in the strawberries treated with *C. citratus* and *C. winterianus* essential oils, and, to a lesser degree, when exposed to *M. alternifolia* essential oil. This behavior is different from those observed by Duque et al.,^51^ who assessed the postharvest quality of tomatoes treated with eugenol (major compound of clove essential oil). However, a similar behavior was observed by Mohammadi and Aminifard,^52^ who evaluated the quality of peaches (*Prunus persica* var. Redhaven) treated with increasing doses of ammi (*Carum copticum*), anise (*Pimpinella anisum*), ziziphora (*Ziziphora clinopodioides*) and cinnamon (*Cinnamomum zeylanicum*) essential oils. Greater essential doses induced higher SS content in the treated peaches. The authors attributed this behavior to a reduced fruit respiration rate induced by the essential oils, which may have helped to conserve metabolic substrates, such as sugars, increasing their content relative to the control, which had a possibly higher respiration rate.

It is also important to observe that there was a no specific relationship between the antifungal and antioxidant effects of the essential oils on the physical-chemical parameters of the tested strawberries. Such a behavior may be the result of the small interaction time between the essential oils and the fruits, of even because the essential oils remain at the fruit surface, not being capable of permeating through the tissues and acting within the fruit.

## 3 Conclusion

The essential oils tested in this study demonstrated a fungicidal effect against both phytopathogens at the highest concentration (0.20% v/v). The essential oil of *C. citratus* showed, at a concentration of 0.05% v/v, total control of the development of *B. cinerea* and *C. acutatum.* It also demonstrated a better proportion (ratio) of soluble solids and titratable acidity, resulting in better fruit flavor, thus providing better productivity and quality of strawberry fruits. It was possible to observe a relationship between antioxidant activity, chemical composition, and antifungal effect, in which the essential oils with the strongest antifungal effect had also the highest antioxidant effect. These results may not only impact product quality but also represent a significant economic aspect for agriculture, which seems promising from the point of view of reducing food waste, reducing the use of synthetic products, and improving the quality and nutritional value of food. In this sense, essential oils may be promising alternative to obtain and conserve fruit products with a better quality and low degree of potential contamination.

## 4 Material and Methods

### 4.1 Extraction of Essential Oils and Analysis of Chemical Composition

The essential oils of *C. citratus* (DC.) Stapf, *R. officinalis* L., *C. winterianus* Jowitt., *M. alternifolia*, and *C. camphora* var. *linaloolifera* were extracted from the dried leaves of plants grown in Caxias do Sul and Nova Petrópolis, Rio Grande do Sul, Brazil.

The essential oil was extracted by steam-distillation for 2 h, in the Laboratory of Soil-Plant Studies (LESPA) of the University of Caxias do Sul/RS. Chromatographic analysis was performed to elucidate the chemical composition at the Agronomic Institute (IAC).

The essential oil samples were diluted in ethyl acetate (Tedia, chromatographic grade, Fairfield, OH, USA, 1.0 g·L^–1^) and 1 μL of the solution was injected. The analyses were performed using a Thermo Scientific gas chromatograph (model TRACE 1300 Series GC—Thermo Scientific—Waltham, MA, USA) equipped with a mass spectrometer (model ISQ 7000), and a Triplus RSH automatic injector (ThermoFisher Scientific). The injector was maintained at 220 °C, with a helium carrier gas flow (99.9999% purity) at a split ratio of 1:20. The mass spectrometer (MS) operated in full scan mode, using electron ionization (70 eV), with an acquisition range from 40 to 450 *m*/*z*. The transfer line and the ion source were operated at 250 and 230 °C, respectively. Separation of the substances was carried out using an Rtx-5MS capillary column (30 m × 0.25 mm, 0.25 μm, Restek Co., Bellefonte, PA, USA), with a carrier gas flow of 1.0 mL.min^–1^ under the following temperature program: 60 to 240 °C at 3.0 °C·min^–1^. The *Chromeleon* software 7 (Thermo Scientific, Waltham, MA, USA) was used for data acquisition and processing. The identification of the substances was performed by comparing the mass spectra with the National Institute of Standards and Technology (NIST 14) library, the Flavor & Fragrance Natural & Synthetic Compounds (FFNSC 3) library, and the linear retention indices (LRI) of the substances with data from the literature.^53^ The linear retention indices were obtained by injecting a series of *n*-alkanes (C_9_–C_24_, Sigma-Aldrich, St. Louis, MI, USA, 99%) under the same chromatographic conditions as the samples, using the Van den Dool and Kratz equation.^54^ The data were expressed as relative percentages based on the area normalization method.

### 4.2 Evaluation of Antioxidant Activity

The antioxidant activity of the oils was evaluated by inhibiting the DPPH^•^ and ABTS^•+^ radicals. The methodology proposed by Santos^50^ was used to evaluate the inhibition of the DPPH^•^ radical. The ability to reduce the ABTS^•+^ radical was determined according to the method proposed by Rufino.^56^

### 4.3 Origin of Isolates

Samples of the phytopathogens causing postharvest diseases in strawberries, *B. cinerea* (MH665643.1) and *C. acutatum* (LR33 OR744907), were taken from the mycotheque of the Phytopathology Laboratory of the University of Caxias do Sul.

### 4.4 Evaluation of the *In Vitro* Antifungal Activity of Essential Oils

To evaluate the effect of essential oils on the mycelial growth of each pathogen, different concentrations (zero; polysorbate 0.20% v/v, 0.01%, 0.05%, 0.10%, 0.15%, and 0.20% v/v) were used, diluted in polysorbate 20 (1:1). All concentrations were diluted in 100 mL of PDA culture medium. The culture media and essential oil were distributed in Petri dishes (20 mL per dish). After solidification, a 0.5 cm diameter disk containing mycelia of the fungus removed from a colony with 14 days of growth was transferred to the center of each dish. In the control and polysorbate treatments, the pathogen was inoculated on plates containing only PDA.^55^ The treatments were incubated in a growth chamber with a photoperiod of 12 h and a temperature of 25 °C. A completely randomized design was used, with ten replicates, each plot consisting of a Petri dish. The mean diameter of the colonies was measured with a digital caliper at three, five, seven, ten, and 14 days. The percentage of growth inhibition (PI) of the treatments was also determined relative to the control.^21^ The best results (considering the lowest possible concentrations) obtained in the *in vitro* test were applied to the *in vivo* test.

### 4.5 Evaluation of the *In Vivo* Antifungal Activity of Essential Oils

Strawberry fruits of the San Andreas variety were purchased from a packing house (Quintal da Alice, Vila Seca, RS). The fruits were superficially disinfected by immersion for 1 min in a sequence of solutions (70% v/v alcohol, 1.0% v/v sodium hypochlorite, and washed in sterile distilled water), as Fontana et al.^1^ proposed.

The antifungal activity of essential oils on strawberry fruits was evaluated according to the method described by Pedrotti,^42^ with modifications. The best concentrations of essential oils in the *in vitro test* (two concentrations of each oil) were mixed with 100 mL of water and the surfactant polysorbate 20 (1:1). The curative test was performed, i.e., a 10 μL aliquot of a suspension with 1·10^5^ spores·mL^–1^ of *C. acutatum*, and 6·10^4^ spores·mL^–1^ of *B. cinerea* in the equatorial region of the fruit. Four hours later, the treatments were sprayed on the fruits and left to dry naturally on sterile paper. The fruits of the control treatment were sprayed with sterile distilled water and polysorbate solution (0.20% v/v).

The experimental design used for the postharvest treatment of strawberries was completely randomized. For *C. acutatum*, there were three treatments, while for *B. cinerea,* there were six treatments and 24 replicates per treatment, with each fruit considered an experimental unit.

The fruits were placed in plastic boxes (30 cm × 10 cm × 13 cm) and incubated at 25 ± 1 °C and 80–90% relative humidity, with a 12 h photoperiod for 5 days. After incubation, the severity of the disease was assessed, and the surface area of decomposition in the fruit was visually evaluated on a scale of zero to 100%, as shown in Figure 5.

**Figure 5:**
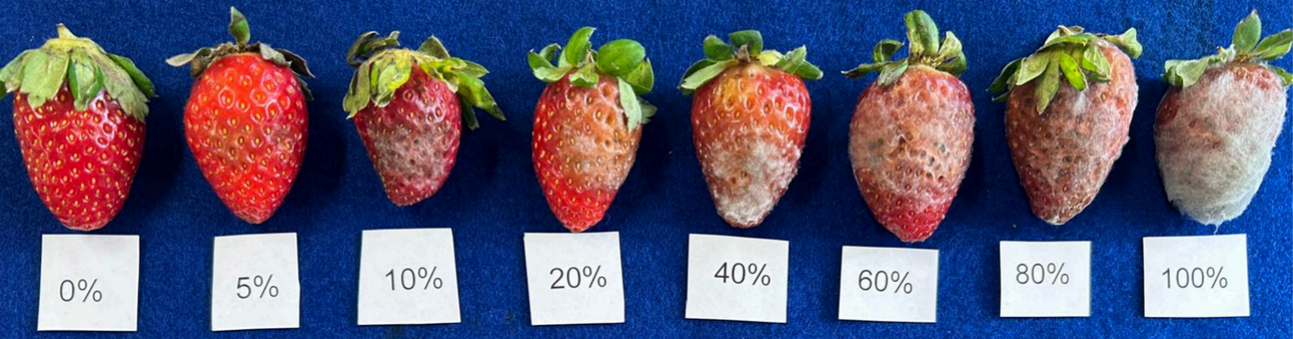
Diagrammatic scale for measuring disease severity in strawberry fruits.^57^

### 4.6 Effect of Selected Essential Oils on Natural Fruit Deterioration and Postharvest Quality

Fruit firmness was assessed using a benchtop penetrometer (Didatica SP) with a flat probe (4 mm diameter). The results were expressed in Newtons (N) and calculated from two penetrations into the distal region of the fruit.

The strawberries were homogenized with a blender (HR1652/90, Philips) without adding water for the other analyses. The soluble solids (SS) content of the fruits was measured with a digital refractometer (Atago - Pocket Pal-1) and expressed in °Brix degrees (°Bx). The pH of the samples was measured using a digital pH meter (Digimed DM-22). Titratable acidity (TA) was determined by neutralization volumetry, using 0.1 M NaOH as titrant and phenolphthalein as an indicator. The results were expressed in gram-equivalents of citric acid per 100 g of fruit.^58^

### 4.7 Experimental Design and Statistical Analysis

The experimental design was completely randomized for all treatments with essential oils, with seven replicates per treatment in the *in vitro* tests and 20 replicates in the *in vivo* tests. The data obtained were subjected to the Levene test (homoscedasticity) and Shapiro-Wilk test (normality of residuals), followed by the analysis of variance (ANOVA). The means were compared by the Tukey test at a 5% probability of error (α = 0.05) using the AgroEstat software (Brazil).

## References

[1] Fontana D. C., Neto D. D., Pretto M. M., Mariotto A. B., Caron B. O., Kulczynski S. M., Schmidt D. (2021). Using essential oils to control diseases
in strawberries and peaches. Int. J. Food Microbiol..

[2] Sharma A., Rajendran S., Srivastava A., Sharma S., Kundu B. (2017). Antifungal
activities of selected essential oils against *Fusarium oxysporum* f, sp, *lycopersici* 1322, with emphasis on *Syzygium aromaticum* essential oil. J. Biosci, Bioeng.

[3] CRESCENTE G., CASCONE G., VOLPE M. G., MOCCIA S. (2024). Application of PLA-Based
Films to Preserve Strawberries’ Bioactive Compounds. Foods.

[4] Pérez-Rojas M., Díaz-Ramírez D., Ortíz-Ramírez C. I., Galaz-Ávalos R. M., Loyola-Vargas V. M., Ferrándiz C., Abraham-Juárez M. d. R., Marsch-Martínez N. (2023). The Role of
Cytokinins during the Development of Strawberry Flowers and Receptacles. Plant.

[5] Veloso J., van Kan J. A. L. (2018). Many shades of
grey in *Botrytis*–host
plant interactions. Trends Plant Sci..

[6] Bi K., Liang Y., Mengiste T., Sharon A. (2023). Killing softly: a roadmap
of *Botrytis cinerea* pathogenicity. Trends Plant Sci..

[7] Hua L., Yong C., Zhanquan Z., Boqiang L., Guozheng Q., Shiping T. (2018). Pathogenic mechanisms
and control strategies of *Botrytis cinerea* causing
postharvest decay in fruits and
vegetables. Food Quality Saf..

[8] Pereira W. V., Primiano I. V., Morales R. G. F., Peres N. A., Amorim L., May De Mio L. L. (2017). Reduced sensitivity to azoxystrobin of *Monilinia
fructicola* isolates from Brazilian stone fruits is not associated
with previously described mutations in the cytochrome b gene. Plant Dis.

[9] Huong L. T., Thinh B. B., Hung N. H., Phu H. V., Hieu N. C., Dai D. N. (2024). Chemical composition,
antimicrobial and larvicidal
activities of essential oils of two *Syzygium* species
from Vietnam. Braz. J. Biol..

[10] MWITHIGA G., MAINA S., GITARI J., MUTURI P. (2022). Rosemary (*Rosmarinus
officinalis* L.) growth rate, oil yield and oil quality under
differing soil amendments. Heliyon.

[11] Rehman R., Hanif M. A., Mushtaq Z., Al-Sadi A. M. (2016). Biosynthesis of
essential oils in aromatic plants: a review. Food Rev, Int..

[12] González-Aguilar, G. A. ; Ruiz-Cruz, S. ; Cruz-Valenzuela, R. ; Ayala-Zavala, J. F. , New Technologies to Preserve Quality of Fresh-cut Produce, In Food Engineering, Integrated Approaches; Springer, New York, pp, 105–115, 2008.

[13] Karaca N., Demirci B., Gavahian M., Demirci F. (2023). Enhanced Bioactivity
of Rosemary, Sage, Lavender, and Chamomile Essential Oils by Fractionation,
Combination, and Emulsification. ACS Omega.

[14] Borges C. D., Mendonça C. R. B., Zambiazi R. C., Nogueira D., Silva E. M. P. D., Paiva F. F. (2013). Conservação de morangos
com revestimentos à base de Goma xantana e óleo essencial
de sálvia. Biosci. J..

[15] Zhang X., Guo Y., Guo L., Jiang H., Ji Q. (2018). *In Vitro* Evaluation
of Antioxidant and Antimicrobial Activities of *Melaleuca alternifolia* Essential Oil. Biomed Res. Int..

[16] Oliveira J., Parisi M. C. M., Baggio J. S., Silva P. P. M., Paviani B., Spoto M. H. F., Gloria E. M. (2019). Control of *Rhizopus stolonifer* in strawberries by the combination of
essential oil with carboxymethylcellulose. Int.
J. Food Microbiol..

[17] de
Carvalho Z. S. (2023). Potencial fitoquímico dos óleos
essenciais: exploração e aplicações. Boletim Científico Agronômico do CCAAB/UFRB.

[18] BOUKHATEM M. N., FERHAT M. A., KAMELI A., SAIDI F., KEBIR H. T. (2014). Lemon grass
(*Cymbopogon citratus*) essential oil as a potent anti-inflammatory
and antifungal drugs. Libyan J. Med..

[19] da
Silva L. C., de Souza Perinotto W.
M., Sá F. A., de Souza M. A. A., de Oliveira Barbosa Bitencourt R., Sanavria A., Santos H. A., Marie-Magdeleine C., da Costa Angelo I. (2020). In vitro acaricidal activity of *Cymbopogon
citratus*, *Cymbopogon nardus* and *Mentha arvensis* against *Rhipicephalus microplus* (Acari: Ixodidae). Exp Parasitol..

[20] BORDIN C., ALVES D. S., ALVES L. F. A., OLIVEIRA M. S., ASCARI J., SCHARF D. R. (2021). Fumigant activity of essential oils
from *Cinnamomum* and *Citrus* spp.
and pure compounds against *Dermanyssus gallinae* (De
Geer) (Acari: Dermanyssidae) and
toxicity toward the non-target organism *Beauveria bassiana* (Vuill.). Vet Parasitol..

[21] Pansera M. R., Silvestre W. P., Sartori V. C. (2022). Bioactivity of *Cupressus
sempervirens* and *Cupressus lusitanica* leaf
essential oils on *Colletotrichum fructicola*. J. Essential Oil Res..

[22] Martins W. d. S., de Araújo J. S.
F., Feitosa B. F., Oliveira J. R., Kotzebue L. R. V., Agostini D. L. d. S., de Oliveira D. L. V., Mazzetto S. E., Cavalcanti M. T., da Silva A. L. (2021). Lemongrass (*Cymbopogon citratus* dc,
stapf) essential oil microparticles:
development, characterization, and antioxidant potential. food chem.

[23] Pansera M. R., Silvestre W. P., Touguuinha L. B. A., Sartori V. C. (2023). Antifungal and Antioxidant
Activity of *Cupressus sempervirens* and *Cupressus
lusitanica* Botanical Fermentates on *Colletotrichum
fructicola*: In Vitro and In Vivo Evaluation. Revista De Gestão Social E Ambiental.

[24] Senol F. S., Orhan I. E., Ustun O. (2015). In vitro cholinesterase
inhibitory
and antioxidant effect of selected coniferous tree species. Asian Pac. J. Trop. Med..

[25] Clain E., Baranauskienė R., Kraujalis P., Šipailienė A., Maždžierienė R., Kazernavičiu̅tė R., El Kalamouni C., Venskutonis P. R. (2018). Biorrefinação de *Cymbopogon nardus* da Ilha da Reunião em óleo
essencial e frações anti oxidantes por métodos
de extração convencionais e de alta pressão. Ind, Culturas Prod.

[26] Paudel P. N., Satyal P., Satyal R., Setzer W. N., Gyawali R. (2022). Chemical Composition,
Enantiomeric Distribution, Antimicrobial and Antioxidant Activities
of *Origanum majorana* L, Essential Oil from Nepal. Molecules.

[27] Yan J., Wu H., Chen K., Feng J., Zhang Y. (2021). Antifungal Activities
and Mode of Action of *Cymbopogon citratus*, *Thymus vulgraris*, and *Origanum heracleoticum* Essential Oil Vapors against *Botrytis cinerea* and
Their Potential Application to Control Postharvest Strawberry Gray
Mold. Foods.

[28] Yan J., Wu H., Shi F., Wang H., Chen K., Feng J., Jia W. (2020). Antifungal activity screening for mint and thyme essential oils against *Rhizopus stolonifer* and their application in postharvest
preservation of strawberry and peach fruits. J, Appl, Microbiol..

[29] Kawhena T.
G., Opara U. L., Fawole O. A. (2021). A Comparative study of antimicrobial
and antioxidant activities of plant essential oils and extracts as
candidate ingredients for edible coatings to control decay in ‘Wonderful’
pomegranate. Molecules.

[30] Xu Y., Tong Z., Zhang X., Wang Y., Fang W., Li L., Luo Z. (2019). Unveiling the mechanisms for the plant volatile organic
com-pound linalool to control gray mold on strawberry fruits. J, Agric, Food Chem..

[31] Li X., Wang Q., Li H., Wang X., Zhang R., Yang X., Jiang Q., Shi Q. (2022). Revealing the Mechanisms
for Linalool Antifungal Activity against *Fusarium oxysporum* and Its Efficient Control of Fusarium Wilt in Tomato Plants. Int. J. Mol. Sci..

[32] Shen Q., Li H., Wang Q., Wang J., Ge J., Yang X., Wang X., Li X., Zhang Y., Zhang R., Shi Q. (2022). Alleviating Effects of Linalool Fumigation on *Botrytis cinerea* Infections in Postharvest Tomato Fruits. Horticulturae.

[33] Anaruma N. D., Schmidt F. L., Duarte M. C. T., Figueira G. M., Delarmelina C., Benato E. A., Sartoratto A. (2010). Control of *Colletotrichum
gloeosporioides* (penz,) Sacc, In yellow passion fruit using *Cymbopogon citratus* essential oil. Braz J. Microbiol..

[34] Oliveira
Filho J. G. d., Albiero B. R., Calisto Í. H., Bertolo M. R. V., Oldoni F. C. A., Egea M. B., Bogusz
Junior S., de Azeredo H. M. C., Ferreira M. D. (2022). Bio-nanocomposite
edible coatings based on arrowroot starch/cellulose nanocrystals/carnauba
wax nanoemulsion containing essential oils to preserve quality and
improve shelf life of strawberry. Int. J. Biol.
Macromol..

[35] Wijesundara N. M., Lee S. F., Cheng Z., Davidson R., Rupasinghe H. P. V. (2021). Carvacrol
exhibits rapid bactericidal activity against *Streptococcus
pyogenes* through cell membrane damage. Sci. Rep.

[36] Kim J., Lee Y., Lee S., Shin S., Park I. (2008). Fumigant antifungal
activity of plant essential oils and components from West Indian bay
(*Pimenta racemosa*) and thyme (*Thymus vulgaris*) oils against two phytopathogenic fungi. Flavour
Fragr..

[37] He J., Wu D., Zhang Q., Chen H., Li H., Han Q., Lai X., Wang H., Wu Y., Yuan J., Dong H., Qin W. (2018). Efficacy and Mechanism of Cinnamon Essential Oil on Inhibition of
Colletotrichum acutatum Isolated From ‘Hongyang’ Kiwifruit. Front Microbiol..

[38] Piechowiak T., Skóra B. (2023). Edible coating
enriched with cinnamon oil reduces the
oxidative stress and improves the quality of strawberry fruit stored
at room temperature. J. Sci. Food Agric..

[39] Venancio A. N., Silva M. J., Parreira L. A., Júlio A. A., Souza G. R., Conceição
Santos M. F., Menini L. (2025). Citronellal: a natural aldehyde with
important properties. Nat. Prod. Res..

[40] Chen W., Viljoen A. M. (2022). Geraniol –
A review update. S. Afr. J. Bot..

[41] Numpaque M. A., Oviedo L. A., Gil J. H., García C. M., Durango D. L. (2011). Thymol and carvacrol:
Biotransformation and antifungal
activity against the plant pathogenic fungi *Colletotrichum
acutatum* and *Botryodiplodia theobromae*. Trop, Plant Pathol.

[42] Pedrotti C., Ribeiro R. T. S., Schwambach J. (2019). Control of
postharvest fungal rots
in grapes through the use of *Baccharis trimera* and *Baccharis dracunculifolia* essential oils. Crop Prot..

[43] Kaur G., Ganjewala D., Bist V., Verma P. C. (2019). Atividades antifúngicas
e larvicidas de dois monoterpenos acíclicos; citral e
geraniol contra fungos e insetos fitopatogênicos. Arco. Fitopatol. Planta. Prot..

[44] Petrasch S., Knapp S. J., van Kan J. A. L., Blanco-Ulate B. (2019). Grey mould
of strawberry, a devastating disease caused by the ubiquitous necrotrophic
fungal pathogen *Botrytis cinerea*. Mol, Plant Pathol.

[45] Pérez-cordero A., Chamorro-anaya L., Vitola-romero D., Hernández-gómez J. (2017). Actividad
antifúngica de *Cymbopogon citratus* contra *Colletotrichum gloeosporioides*. Agron,
Mesoam.

[46] Tofiño-Rivera A., Ortega-Cuadros M., Galvis-Pareja D., Jiménez-Rios H., Merini L. J., Martínez-Pabón M. C. (2016). Efeito
dos óleos essenciais *de Lippia alba* e *Cymbopogon citratus em biofilmes de Streptococcus mutans* e citotoxicidade em células CHO. J,
Etnofarmacol.

[47] CARRILLO, JH ; ZAVALZA E, O ; RODŔGUEZ, SG ; C TORRES, CM ; AHUMADA, DS ; RIVERA, YO . Evaluation of the effectivity of reuterin in pectin edible coatings to extend the shelf-life of strawberries during cold storage, Food Packag, Shelf Life 2021, 30.

[48] Liu Z., Liang T., Kang C. (2023). Molecular
bases of strawberry fruit
quality traits: Advances, challenges, and opportunities. Plant Physiol.

[49] Zulim
Leite A. R., Reitz Cardoso F. A., Correia Gardenal A., de Mello J. C. P., Medeiros Marques L. L., Ferreira Geraldo Perdoncini M. R. (2022). Control
of fungal spoilage in strawberries using crude plant extracts against
the fungus *Botrytis cinerea*. Natural Product Research.

[50] Farnezi P. K. B., Oliveira L. L. d., Sardinha L. T., França A. C., Machado C. M. M., Macedo L. A. (2020). Produção e caracterização
fisico-quimica de morango (*Fragaria* X *Ananassa* Duch) sob diferentes fontes de adubação fosfatada/Production
and physical and chemical characterization of strawberry (*Fragaria* X *Ananassa* Duch) under different
sources of phosphate fertilization. Brazilian
Journal of Development.

[51] Duque L. F., Amador M. V., Guzmán M., Asensio C., Valenzuela J. L. (2021). Development
of a New Essential Oil-Based Technology to Maintain Fruit Quality
in Tomato. Horticulturae.

[52] Mohammdi S., Aminifard M. H. (2012). Effect
of Essential Oils on Postharvest Decay and Some
Quality Factors of Peach (Prunus persica var. Redhaven). J. BIOL. ENVIRON. SCI..

[53] Adams, R. P. Identification of Essential Oil Components by Gas Chromatography/Mass Spectrometry, 5th ed.; Texensis Publishing: Gruver, TX, USA, 2017; pp 46–52.

[54] van
Den Dool H., Dec. Kratz P. (1963). A generalization of the retention
index system including linear temperature programmed gas-liquid partition
chromatography. J. Chromatogr..

[55] Triaca T., Pansera M. R., Andreolla M. L., Venturin L., Sartori V. C. (2018). Avaliação
in vivo do fermentado botânico de *Ilex paraguariensis* frente ao fungo *Sclerotinia sclerotiorum* no cultivo
de alface crespa. Pesquisa Aplicada & Agrotecnologia,
Guarapuava-PR.

[56] Rufino M. D. S. M., Alves R. E., de Brito S. M., Sampaio C. D. G., Pérez-Jimenez J., Saura-Calixto F. D. (2007). Metodologia
Científica: Determinação
da Atividade Antioxidante Total em Frutas pela Captura do Radical
Livre ABTS+. Embrapa Agroindústria Tropical.
Comunicado Técnico.

[57] Arias S. M., Álvarez G. E. G., Patiño P. A. G. (2020). Diagrammatic scale for measuring
severity of gray mould in thornless Castilla blackberry (*Rubus
glaucus* Benth). Ciência Rural.

[58] RICHTER A. (2018). Produtividade e qualidade de cultivares de morangueiro
sob cultivo
de solo e semi-hidropônico. Revista Científica
Rural.

